# Ethoxyquin – Bestimmung von 2,2,4-Trimethyl-6(2H)-chinolinon in Urin mittels UPLC-MS/MS: Biomonitoring-Methode

**DOI:** 10.34865/bi9153d11_2or

**Published:** 2026-06-30

**Authors:** Gerhard Scherer, Nikola Pluym, Max Scherer, Nadine Rögner, Markus Stoeckelhuber, Thomas Jäger, Thomas Göen, Andrea Hartwig, MAK Commission

**Affiliations:** 1 ABF Analytisch-biologisches Forschungslabor GmbH Semmelweisstraße 5 82152 Planegg Deutschland; 2 BASF SE, Corporate Health Management Carl-Bosch-Straße 38 67056 Ludwigshafen Deutschland; 3 Friedrich-Alexander-Universität Erlangen-Nürnberg, Institut und Poliklinik für Arbeits-, Sozial- und Umweltmedizin Henkestraße 9–11 91054 Erlangen Deutschland; 4 Institut für Angewandte Biowissenschaften, Abteilung Lebensmittelchemie und Toxikologie, Karlsruher Institut für Technologie (KIT) Adenauerring 20a, Geb. 50.41 76131 Karlsruhe Deutschland; 5 Ständige Senatskommission zur Prüfung gesundheitsschädlicher Arbeitsstoffe, Deutsche Forschungsgemeinschaft, Kennedyallee 40, 53175 Bonn, Deutschland. Weitere Informationen: Ständige Senatskommission zur Prüfung gesundheitsschädlicher Arbeitsstoffe | DFG

**Keywords:** Ethoxyquin, EQI, Hydroxyquin, Biomonitoring, Urin, UPLC-MS/MS

## Abstract

The working group “Analyses in Biological Materials” of the German Senate Commission for the Investigation of Health Hazards of Chemical Compounds in the Work Area (MAK Commission) developed and verified this biomonitoring method for the determination of the most important urinary metabolite of ethoxyquin – 2,2,4‑trimethyl‑6(2H)-quinolinone (EQI). Ethoxyquin is a quinoline-based synthetic antioxidant. Its use as active substance in pesticides was prohibited in 2011. Nevertheless, it continues to be used as a feed additive and is mainly introduced into the environment by feeding treated fishmeal to fish in aquaculture. A human metabolism study showed that ethoxyquin is first metabolised to 1,2‑dihydro-2,2,4‑trimethyl-6‑quinolinol (hydroxyquin) and is then further oxidised to the more stable EQI. In the biomonitoring method presented here, the glucuronide of hydroxyquin in the urine sample is enzymatically hydrolysed using *β*‑glucuronidase from *E. coli*. Thereafter, hydroxyquin oxidises spontaneously to EQI. The hydrolysate is subject to salt-assisted liquid-liquid extraction (SALLE) with ethyl acetate. Analysis is performed by UPLC‑MS/MS after positive electrospray ionisation (ESI+). Calibration is performed using standards prepared in pooled urine and processed analogously to the samples. EQI‑D_10_ is applied as an internal standard. The method provides reliable and accurate analytical results, as shown by the good precision data with standard deviations no greater than 6%. Good accuracy data were obtained with mean relative recoveries in the range of 103–110%. The method is both selective and sensitive, whereby a quantitation limit of 0.03 μg EQI/l was achieved.

## 1 Kenndaten der Methode

### Matrix und analytisches Messprinzip

**Table:** 

**Matrix**	Urin
**Analytisches Messprinzip**	Ultra-Hochleistungsflüssigkeitschromatographie mit Tandem-Massenspektrometrie (UPLC‑MS/MS)

### Parameter und entsprechender Arbeitsstoff

**Table:** 

Arbeitsstoff	CAS‑Nr.	Parameter	CAS‑Nr.
Ethoxyquin	91-53-2	2,2,4‑Trimethyl-6(2H)‑chinolinon (EQI)	4071-18-5

### Zuverlässigkeitskriterien

#### 2,2,4‑Trimethyl-6(2H)‑chinolinon (EQI)

**Table:** 

Präzision in der Serie:	Standardabweichung (rel.)	*s_w_* = 5,72 %, 0,51 %, 2,28 % bzw. 1,71 %
	Streubereich	*u* = 15,8 %, 1,42 %, 6,33 % bzw. 4,75 %
	bei einer dotierten Konzentration von 0,03 μg, 0,1 μg, 1,0 μg oder 10 μg EQI pro Liter Urin und n = 5 Bestimmungen	
Präzision von Tag zu Tag:	Standardabweichung (rel.)	*s_w_* = 5,34 %, 3,53 %, 3,13 % bzw. 3,44 %
	Streubereich	*u* = 23,0 %, 15,2 %, 13,5 % bzw. 14,8 %
	bei einer dotierten Konzentration von 0,03 μg, 0,1 μg, 1,0 μg oder 10 μg EQI pro Liter Urin und n = 3 Bestimmungen	
Richtigkeit:	Wiederfindung (rel.)	*r* = 106 %, 107 %, 110 % bzw. 103 %
	bei einer dotierten Konzentration von 0,03 μg, 0,1 μg, 1,0 μg oder 10 μg EQI pro Liter Urin und n = 3 Bestimmungen	
Nachweisgrenze:	0,01 μg EQI pro Liter Urin	
Bestimmungsgrenze:	0,03 μg EQI pro Liter Urin	

## 2 Allgemeine Informationen zu Ethoxyquin

Ethoxyquin (6-Ethoxy-2,2,4-trimethyl-1,2-dihydrochinolin) ist ein synthetisches Antioxidans aus der Stoffgruppe der Chinoline. Ethoxyquin war als biozider Wirkstoff in der Europäischen Union zugelassen. Die Verwendung von Ethoxy­quin in Pflanzenschutzmitteln ist jedoch seit Ende 2011 in der Europäischen Union verboten (Europäische Kommission 2011). Ethoxyquin wurde weiterhin als Futtermittelzusatzstoff verwendet, um Futtermittel länger haltbar zu machen und Fischmehl beim Transport auf dem Seeweg vor Selbstentzündung zu schützen (EFSA FEEDAP Panel 2015). Da allerdings nach wie vor keine vollständige Expositionsbewertung für Ethoxyquin vorliegt, wurde die Zulassung von Ethoxy­quin als Futtermittelzusatzstoff von der Europäischen Kommission zum 30.09.2019 ausgesetzt (Europäische Kommission 2017). Im Jahr 2022 hat die EFSA Ethoxyquin als Futtermittelzusatzstoff neu bewertet (EFSA FEEDAP Panel et al. 2022). Ethoxy­quin wird aus *p*-Phenitidin (4-Ethoxyanilin) synthetisiert, das als potentielles Mutagen eingestuft ist und in Konzentrationen von < 2,5 mg/kg Ethoxyquin im Produkt vorliegt. Aufgrund fehlender Daten über das Vorhandensein von *p*-Phenetidin in Lebensmitteln tierischen Ursprungs konnte Ethoxyquin hinsichtlich der Verbrauchersicherheit nicht abschließend bewertet werden. Es wurde jedoch empfohlen, die inhalative Exposition gegen Ethoxyquin aufgrund dieser Verunreinigung so gering wie möglich zu halten. 2022 trat außerdem eine Durchführungsverordnung in Kraft, die die Zulassung von Ethoxyquin als Futtermittelzusatzstoff der Funktionsgruppe „Antioxidationsmittel“ in der Europäischen Union verweigert (Europäische Kommission 2022). Ethoxyquin wurde von der MAK-Kommission bisher nicht bewertet.

Ethoxyquin gelangt hauptsächlich über Fischfarmen in die Umwelt, in denen Speisefische, wie beispielsweise Lachs, mit ethoxiquinversetztem Fischmehl gefüttert werden. Für Fische jeglicher Art existieren bislang keine Grenzwerte. Bei einer von Greenpeace 2016 durchgeführten Studie wurden in insgesamt 54 Fischprodukten aus deutschen Lebensmittelgeschäften bis zu 881 μg Ethoxyquin/kg detektiert (Greenpeace 2016). Ethoxyquin kann sowohl im Fischfutter als auch im Fisch selbst in ein Dimer umgewandelt werden, wobei sich dieses Ethoxyquindimer vor allem im Fettgewebe anreichert (Ørnsrud et al. 2011).

Der Metabolismus von Ethoxyquin wurde insbesondere in Wasserorganismen umfassend untersucht. Die Verstoffwechselung in Fischen erfolgt vor allem durch O‑Deethylierung am C6 zum 1,2‑Dihydro-2,2,4‑trimethyl-6‑chinolinol (Hydroxyquin) und anschließende Konjugation zum Sulfat oder Glucuronid. Auch eine Oxidation des Hydroxyquins zu 2,2,4‑Trimethyl-6(2H)‑chinolinon (EQI) wurde nachgewiesen. Des Weiteren sind Hydroxylierung und Glucuronidierung am C8, Epoxidierung zwischen C3 und C4 und Dimerisierung beobachtet worden (Berdikova Bohne et al. 2006, 2007, 2008; Burka et al. 1996; Kranawetvogl und Elsinghorst 2019). Für lange Zeit war der Metabolismus von Ethoxyquin im Menschen weitgehend unbekannt. Wichtige Metaboliten sowie deren Toxikokinetik und metabolische Konversionsfaktoren wurden im Rahmen eines Kooperationsprojekts von Bundesumweltministerium (BMU) und dem Verband der chemischen Industrie (VCI) zur Entwicklung von Biomonitoring-Verfahren in einer kleinen Metabolismusstudie mit fünf Freiwilligen ermittelt (Stoeckelhuber et al. 2020). Die Metabolismusstudie umfasste eine einmalige orale Dosis von 0,005 mg Ethoxyquin/kg Körpergewicht. Im Urin der Probanden wurden EQI und das Glucuronid von Hydroxyquin als Hauptmetaboliten des Ethoxyquins identifiziert. Das durch enzymatische Hydrolyse freigesetzte Hydroxyquin wird schnell zu EQI oxidiert. Um diese Oxidation zu verhindern wurde Ascorbinsäure zugesetzt. Diese wird jedoch durch die spätere Extraktion weitestgehend entfernt, sodass 6-OH-EQ nach erfolgter Extraktion quantitativ zu EQI oxidiert wird. Eine weitere Oxidation zum *N*-Oxid wurde nicht beobachtet. Im Laufe der Methodenentwicklung wurde speziell darauf geachtet, das vorliegende Gleichgewicht komplett auf die Seite des EQI zu verschieben. Nach erfolgter Aufarbeitung wurde 6-OH-EQ in den Proben nur noch in Spuren (unterhalb der Bestimmungsgrenze) nachgewiesen. Ethoxyquin selbst wurde nur in geringen Mengen (Anteil < 5 % der Ethoxyquindosis) und lediglich in den hochkonzentrierten Urinproben der Probanden gefunden. Innerhalb von 48 h wurden 28,5 % der oral verabreichten Ethoxyquindosis in Form von EQI mit dem Urin ausgeschieden. Ethoxyquindimere konnten in der Metabolismusstudie in keiner der Proben nachgewiesen werden.

**Abb. 1:**
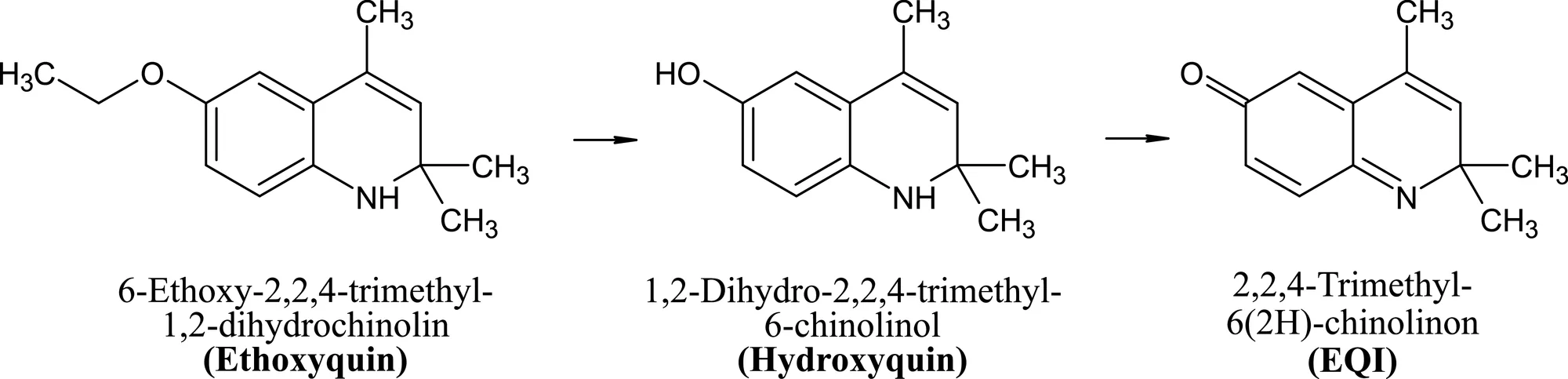
Postulierter Metabolismus von Ethoxyquin im Menschen

Abbildung 1 zeigt die Strukturformel und das postulierte Metabolismusschema von Ethoxyquin im Menschen.

Um die Daten in der Metabolismusstudie zu erheben, haben Stoeckelhuber et al. (2020) eine Biomonitoringmethode entwickelt, die es erlaubt, die Exposition der Allgemeinbevölkerung gegen Ethoxyquin zu erfassen. Bei dieser Methode wird Hydroxyquin während der Aufarbeitung zum stabileren EQI oxidiert, so dass beide Metaboliten gemeinsam als EQI im Urin bestimmt werden. In Tabelle 1 sind exemplarische Hintergrundkonzentrationen von EQI im Urin der deutschen Allgemeinbevölkerung aufgeführt.

**Tab. 1:** EQI im Urin der beruflich nichtexponierten Allgemeinbevölkerung

Studienkollektiv (Land; n; Probe)	QF [%]	NWG [μg/l]	BG [μg/l]	Mittelwert (SD) [μg/l]	GM [μg/l]	Median [μg/l]	P95 [μg/l]	Min–Max [μg/l]	Literatur
Deutschland; 53; Spontanurin	79	0,01	0,03	0,072^a)^ (0,243)^a)^	n. a.	0,015^a)^	n. a.	0,015–1,73	Stoeckelhuber et al. 2020
Deutschland; 250; 24 h-Urin	91	0,01	0,03	0,858^a)^	0,173^a)^	0,16^a)^	1,34^a)^	0,015–104	Pluym et al. 2023

BG: Bestimmungsgrenze; GM: geometrischer Mittelwert; n: Probenanzahl; n. a.: nicht angegeben; NWG: Nachweisgrenze; P95: 95. Perzentil; QF: Bestimmungshäufigkeit (*quantitation frequency*, gemessene Werte oberhalb der BG); SD: Standardabweichung (*standard derivation*)

^a)^ unter Verwendung von BG/2 für Werte unterhalb der BG berechnet

## 3 Grundlage des Verfahrens

Mit dem hier beschriebenen Verfahren wird der Ethoxyquinmetabolit EQI in Urin bestimmt. Der Ethoxyquinmetabolit Hydroxyquin liegt im Urin fast ausschließlich als Glucuronid vor. Nach enzymatischer Hydrolyse mit *β*‑Glucuronidase aus *E. coli* wird das freigesetzte Hydroxyquin spontan zum stabileren EQI oxidiert. Vor der Hydrolyse wird den Proben Ascorbinsäure als Antioxidans zugesetzt, um EQI zu stabilisieren. Zudem wird, um die Effektivität des Hydrolyseschritts zu überprüfen, jeder Probe vor der Hydrolyse 4-Methylumbelliferyl-*β*‑D‑glucuronid (MUG) zugesetzt. Bei erfolgreicher enzymatischer Hydrolyse wird MUG zu 4-Methylumbelliferon (MU) dekonjugiert und nach chromatographischer Trennung massenspektrometrisch detektiert. Das Hydrolysat wird einer salzunterstützten Flüssig-Flüssig-Extraktion (*salt-assisted liquid-liquid extraction*, SALLE) mit Ethylacetat unterzogen. Die Analyse erfolgt mittels UPLC‑MS/MS nach positiver Elektrospray-Ionisierung (ESI+). Die Kalibrierung erfolgt mit Standards, die in Poolurin angesetzt und analog zu den zu analysierenden Proben aufgearbeitet werden. Als interner Standard wird EQI‑D_10_ verwendet.

## 4 Geräte, Chemikalien und Lösungen

### 4.1 Geräte

- UPLC‑Anlage bestehend aus einer Hochdruckpumpe (LC‑20AB), einer Ultradruckpumpe (LC‑30AD), einem System-Controller (CBM‑20A), einem Degasser (DGU‑20A5R), einem Säulenofen (CTO‑20AC) und einem Autosampler (SIL‑30ACMP) (z. B. Shimadzu Deutschland GmbH, Duisburg)
- Tandem-Quadrupol-Massenspektrometer (z. B. QTRAP^®^ 6500+, AB SCIEX Germany GmbH, Darmstadt) mit Software für die Datenauswertung (z. B. Multiquant V.3.0.2, AB SCIEX Germany GmbH, Darmstadt)
- Stickstoffgenerator (z. B. NGM 11‑s, cmc Instruments GmbH, Eschborn)
- HPLC‑Säule (z. B. Nr. 186002352ivd, ACQUITY UPLC BEH C18 1,7 μm, 2,1 × 100 mm, Waters GmbH, Eschborn)
- Reinstwasseranlage (z. B. Sartorius arium^®^, Sartorius AG, Göttingen)
- Pipetten: 1–10 μl, 10–100 μl, 100–1000 μl, 1000–5000 μl (z. B. Varipetten^®^, Eppendorf AG, Hamburg)
- Multipette^®^ (z. B. Eppendorf AG, Hamburg)
- 4‑ml-Gewindeglasflaschen (z. B. Nr. 130400, BGB Analytik Vertrieb GmbH, Rheinfelden) mit Schraubkappen (z. B. Nr. 2.301158, Klaus Ziemer GmbH, Langerwehe)
- 100‑ml-Braunglasflaschen (z. B. Nr. 704012 BRAND GmbH + CO KG, Wertheim)
- Microvials mit Inserts (z. B. Klaus Ziemer GmbH, Langerwehe)
- Verschiedene Messkolben (z. B. Schott AG, Mainz)
- pH‑Meter mit pH‑Elektrode (z. B. Typ CG 842, Schott AG, Mainz)
- Zentrifuge (z. B. Rotixa KS, Andreas Hettich GmbH & Co. KG, Tuttlingen)
- Brutschrank mit Schüttelapparat (z. B. Incucell 111 mit Schüttler GFL 3005, MMM Medcenter Einrichtungen GmbH, Planegg)
- Analysenwaage (z. B. Sartorius AG, Göttingen)
- Multi-Tube Vortex-Mischer (z. B. VWR International GmbH, Darmstadt)
- Vortex-Schüttler (z. B. VWR International GmbH, Darmstadt)
- Crimpzange (z. B. Klaus Ziemer GmbH, Langerwehe)
- Urinbecher (z. B. VWR International GmbH, Darmstadt)

### 4.2 Chemikalien

Wenn nicht anders angegeben, sind alle genannten Chemikalien mindestens in p. a.‑Qualität zu verwenden.

- Acetonitril, LiChrosolv^®^ (z. B. Nr. 10003, Supelco, Merck KGaA, Darmstadt)
- Ameisensäure, 0,1 % in Acetonitril (z. B. Nr. 2645, Th. Geyer GmbH & Co. KG, Renningen)
- Ameisensäure, 99 % (z. B. Nr. 069141, Biosolve B.V., Valkenswaard, Niederlande)
- Ascorbinsäure (z. B. Nr. A5960, Merck KGaA, Darmstadt)
- Essigsäure (Eisessig), > 99 % (z. B. Nr. 33252, Alfa Aesar, Thermo Fisher Scientific^TM^, Life Technologies GmbH, Darmstadt)
- Ethylacetat (z. B. Nr. 2219, Th. Geyer GmbH & Co. KG, Renningen)
- *β*‑Glucuronidase aus *E. coli* (500 kU; 250 kU/ml) (z. B. Nr. E‑BGLAEC, Megazyme Ltd., Bray, Irland)
- Hochreines Wasser (z. B. Sartorius arium^®^, Sartorius AG, Göttingen)
- Magnesiumsulfat (z. B. Nr. M7506, Merck KGaA, Darmstadt)
- Methanol (z. B. Nr. 1428, Th. Geyer GmbH & Co. KG, Renningen)
- 4‑Methylumbelliferyl-*β*‑D‑glucuronid-Dihydrat (z. B. Nr. sc-284360, Santa Cruz Biotechnology Inc., Dallas, USA)
- Natriumchlorid (z. B. Nr. S7653, Merck KGaA, Darmstadt)
- Natriumhydroxid-Plätzchen (z. B. Nr. 106469, Merck KGaA, Darmstadt)
- 2,2,4‑Trimethyl-6(2H)‑chinolinon (EQI) (z. B. Eigensynthese, ABF GmbH, München, siehe Supplementary information zu Stoeckelhuber et al. 2020)
- 2,2,4‑Trimethyl-6(2H)‑chinolinon‑D_10_ (EQI‑D_10_) (z. B. Auftragssynthese, Synthèse AptoChem Inc., Montreal, Kanada)

### 4.3 Lösungen

- Ascorbinsäurelösung (100 g/l)
  In einen 100‑ml-Messkolben werden 10 g Ascorbinsäure eingewogen und in etwas hochreinem Wasser gelöst. Anschließend wird der Messkolben bis zur Markierung mit hochreinem Wasser aufgefüllt.

Die Lösung ist bei Raumtemperatur drei Monate stabil.

- Acetatpuffer (1 mol/l, pH = 5,1)
  In einen 1000‑ml-Messkolben werden etwa 500 ml hochreines Wasser vorgelegt und 57 ml Essigsäure (> 99 %) zugegeben. Der pH‑Wert wird mit Natriumhydroxidplätzchen auf pH = 5,1 eingestellt. Anschließend wird der Messkolben bis zur Markierung mit hochreinem Wasser aufgefüllt.

Die Lösung ist bei Raumtemperatur drei Monate stabil.

- *β*‑Glucuronidase-Suspension (250 kU/ml)
  Ein Fläschchen (2 ml) *β*‑Glucuronidase (500 kU/ml) wird mit 2 ml hochreinem Wasser verdünnt.

Die Lösung ist bei 4 °C drei Monate stabil.

- Natronlauge (14,3 mol/l bzw. 40 %)
  In einem Becherglas wird hochreines Wasser vorgelegt und 57,2 g Natriumhydroxidplätzchen werden darin gelöst. Die Natronlauge wird in einen 100‑ml-Messkolben überführt und dieser bis zur Markierung mit hochreinem Wasser aufgefüllt.

Die Lösung ist bei Raumtemperatur für zehn Jahre stabil.

- Eluent A (0,1 % Ameisensäure in hochreinem Wasser)
  In einen 1000‑ml-Messkolben werden etwa 500 ml hochreines Wasser vorgelegt und 1 ml Ameisensäure wird zugegeben. Anschließend wird der Messkolben bis zur Markierung mit hochreinem Wasser aufgefüllt.

Die Lösung ist bei Raumtemperatur für zehn Jahre stabil.

- Eluent B (0,1 % Ameisensäure in Acetonitril)
  In einen 1000‑ml-Messkolben werden etwa 500 ml Acetonitril vorgelegt und 1 ml Ameisensäure wird zugegeben. Anschließend wird der Messkolben bis zur Markierung mit Acetonitril aufgefüllt.

Die Lösung ist bei Raumtemperatur für zehn Jahre stabil.

- 4‑Methylumbelliferyl-*β*‑D‑glucuronid (MUG)-Stammlösung (5000 mg/l)
  In einen 5‑ml-Messkolben werden 9,5 mg MUG-Dihydrat eingewogen und in 1724 μl Methanol gelöst.

Die MUG-Stammlösung ist bei 4 °C über mehrere Jahre stabil.

- MUG-Arbeitslösung (50 mg/l)
  In einen 10‑ml-Messkolben werden 100 μl der MUG-Stammlösung pipettiert. Anschließend wird der Kolben bis zur Markierung mit hochreinem Wasser aufgefüllt.

Die MUG-Arbeitslösung ist bei 4 °C über mehrere Jahre stabil.

### 4.4 Interner Standard (ISTD)

- ISTD‑Stammlösung (1 g/l)
  In einen 10‑ml-Messkolben werden 10,0 mg EQI‑D_10_ eingewogen und in Acetonitril gelöst. Der Kolben wird anschließend mit Acetonitril bis zur Markierung aufgefüllt.
- ISTD‑Arbeitslösung I (100 mg/l)
  Zu 300 μl ISTD‑Stammlösung werden 2700 μl Acetonitril pipettiert.
- ISTD‑Arbeitslösung II (10 mg/l)
  Zu 300 μl ISTD‑Arbeitslösung I werden 2700 μl Acetonitril pipettiert.
- ISTD‑Dotierlösung (1 mg/l)
  Zu 300 μl ISTD‑Arbeitslösung II werden 2700 μl Acetonitril pipettiert.

Die ISTD-Stammlösung, die ISTD-Arbeitslösungen sowie die ISTD-Dotierlösung werden in Braunglasflaschen bei −20 °C gelagert und sind unter diesen Bedingungen mindestens 18 Monate stabil.

### 4.5 Kalibrierstandards

- EQI-Stammlösung I (1000 mg/l)
  In einen 10‑ml-Messkolben werden 10,0 mg EQI eingewogen und in Acetonitril gelöst. Der Kolben wird anschließend mit Acetonitril bis zur Markierung aufgefüllt.
- EQI-Stammlösung II (100 mg/l)
  Zu 300 μl EQI-Stammlösung I werden 2700 μl Acetonitril pipettiert.
- EQI-Stammlösung III (10 mg/l)
  Zu 300 μl EQI-Stammlösung II werden 2700 μl Acetonitril pipettiert.
- EQI-Arbeitslösung I (1 mg/l)
  Zu 300 μl EQI-Stammlösung III werden 2700 μl Acetonitril pipettiert.
- EQI-Arbeitslösung II (100 μg/l)
  Zu 300 μl EQI-Arbeitslösung I werden 2700 μl Acetonitril pipettiert.
- EQI-Arbeitslösung III (10 μg/l)
  Zu 300 μl EQI-Arbeitslösung II werden 2700 μl Acetonitril pipettiert.
- EQI-Arbeitslösung IV (1 μg/l)
  Zu 300 μl EQI-Arbeitslösung III werden 2700 μl Acetonitril pipettiert.

Die Stamm- und Arbeitslösungen werden in Braunglasflaschen bei −20 °C gelagert. Die EQI-Stammlösung I ist bei −20 °C mindestens 18 Monate stabil.

Für die Kalibrierung wird Poolurin mit möglichst geringen Analytkonzentrationen verwendet. Für die Herstellung der Kalibrierstandards wird der Urin nach dem in Tabelle 2 dargestellten Pipettierschema mit den EQI-Arbeitslösungen und der ISTD-Dotierlösung versetzt. Zur Ermittlung des Leerwerts wird undotierter Poolurin mitgeführt.

**Tab. 2:** Pipettierschema zur Herstellung der Kalibrierstandards für die Bestimmung von EQI in Urin

Kalibrierstandard	EQI-Arbeitslösung IV [μl]	EQI-Arbeitslösung III [μl]	EQI-Arbeitslösung II [μl]	EQI-Arbeitslösung I [μl]	ISTD-Dotierlösung [μl]	Poolurin [μl]	Analytkonzentration [μg/l]
S00	–	–	–	–	–	3000	0
S0	–	–	–	–	10	2990	0
S1	90	–	–	–	10	2900	0,03
S2	–	15	–	–	10	2975	0,05
S3	–	30	–	–	10	2960	0,1
S4	–	60	–	–	10	2930	0,2
S5	–	–	15	–	10	2975	0,5
S6	–	–	30	–	10	2960	1
S7	–	–	60	–	10	2930	2
S8	–	–	–	15	10	2975	5
S9	–	–	–	30	10	2960	10
S10	–	–	–	60	10	2930	20

## 5 Probenahme und Probenaufbereitung

### 5.1 Probenahme

Die Urinproben werden in verschließbaren Urinbechern gesammelt und bis zur Probenaufbereitung bei −20 °C gelagert.

### 5.2 Probenaufbereitung

Die tiefgefrorenen Urinproben werden vor der Analyse bei Raumtemperatur aufgetaut und gut durchmischt. Aliquote von je 3 ml Urin werden in 4‑ml-Gewindeglasfläschchen überführt, anschließend werden 10 μl Ascorbinsäurelösung, 10 μl ISTD-Dotierlösung, 0,5 ml Acetatpuffer, 10 μl MUG‑Arbeitslösung und 10 μl *β‑*Glucuronidase-Suspension zugegeben. Die Proben werden gut gemischt und für eine Stunde in einem Brutschrank bei 37 °C auf einem Vortex-Schüttler bei 100 rpm geschüttelt.

Die hydrolysierten Proben werden vorsichtig in neue 4‑ml-Gläschen überführt, die 0,8 g Magnesiumsulfat und 0,2 g Natriumchlorid enthalten. Anschließend werden 100 μl Natronlauge (14,3 mol/l) und 400 μl Ethylacetat hinzupipettiert. Mit Hilfe eines Multi-Tube Vortex-Mischers werden die Proben 10 min bei 2500 rpm gemischt und anschließend 10 min bei 2500 × *g* zentrifugiert. Proben, bei denen die Phasentrennung unzureichend ist, werden nochmal einzeln geschüttelt und erneut zentrifugiert. Von der organischen Phase werden 100 μl abgehoben, in ein Mikrovial mit Insert überführt und zur Analyse mittels UPLC‑MS/MS eingesetzt.

## 6 Instrumentelle Arbeitsbedingungen

Die analytische Messung erfolgte an einer Gerätekonfiguration bestehend aus UPLC‑Anlage gekoppelt mit einem Tandem-Massenspektrometer.

### 6.1 Flüssigkeitschromatographie

**Table:** 

Trennsäule:	ACQUITY UPLC BEH C18 (2,1 mm × 100 mm, 1,7 μm)
Säulentemperatur:	isotherm, 40 °C
Autosamplertemperatur:	10 °C
Injektionsvolumen:	5 μl
Eluent:	A: Wasser + 0,1 % Ameisensäure
	B: Acetonitril + 0,1 % Ameisensäure
Flussrate:	0,5 ml/min
Druck:	600–700 bar
Gradientenprogramm:	siehe Tabelle 3

**Tab. 3:** Gradientenprogramm für die Bestimmung von EQI in Urin

Zeit [min]	Eluent A [%]	Eluent B [%]
0,00	90	10
1,00	90	10
4,00	10	90
6,00	10	90
6,01	90	10
8,00	90	10

### 6.2 Tandem-Massenspektrometrie

**Table:** 

Ionisierung:	positive Elektrosprayionisierung (ESI+)
Weitere Einstellungen:	siehe Tabelle 4

**Tab. 4:** Massenübergänge, Retentionszeiten sowie weitere parameterspezifische Einstellungen für die Bestimmung von EQI in Urin

Analyt bzw. ISTD	Retentionszeit [min]	Massenübergang (*m/z*)	Declustering-Potenzial [V]	Kollisionsenergie [V]	Kollisionszellen-Austrittspotenzial [V]
MU	2,9	177,1 → 76,9^a)^	36	49	10
EQI	3,4	188,0 → 173,1^a)^	13	21	10
	3,4	188,0 → 145,1	13	35	10
EQI-D_10_	3,3	198,0 → 152,1^a)^	13	35	10

EQI: 2,2,4‑Trimethyl-6(2H)‑chinolinon; ISTD: interner Standard; MU: 4-Methylumbelliferon

^a) ^Quantifier

Die gerätespezifischen Parameter müssen vom Anwender individuell für die eingesetzten Geräte ermittelt und eingestellt werden. Die in diesem Abschnitt genannten gerätespezifischen Parameter sind für das während der Methodenentwicklung verwendete System bestimmt und optimiert worden.

**Abb. 2:**
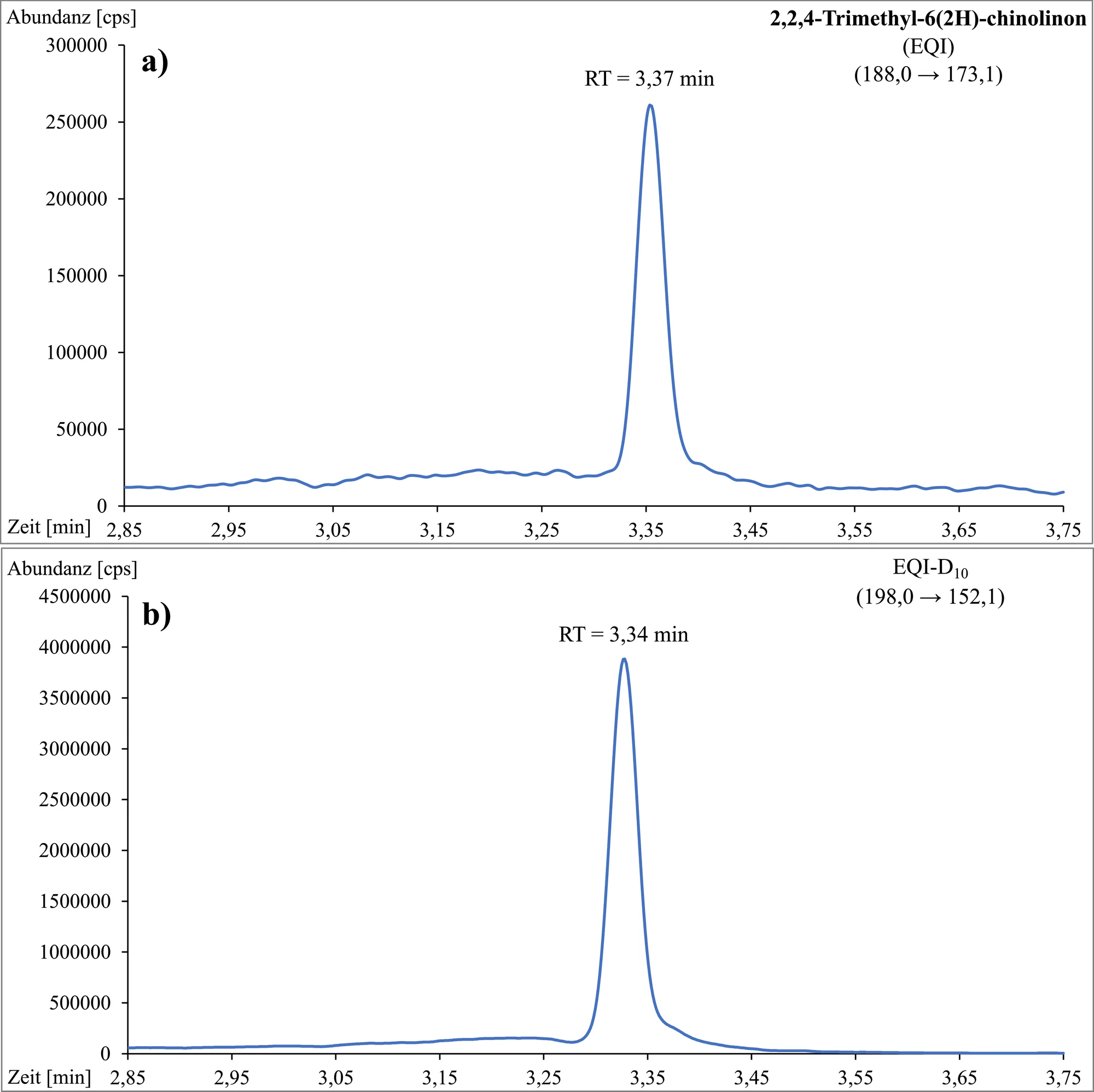
Chromatogramm einer mit 0,1 μg EQI pro Liter dotierten Urinprobe

Abbildung 2 zeigt ein repräsentatives Chromatogramm einer mit 0,1 μg EQI/l dotierten Urinprobe.

## 7 Analytische Bestimmung

Zur analytischen Bestimmung der nach Abschnitt 5 aufgearbeiteten Urinproben wird ein Aliquot von 5 μl mittels Autosampler in das UPLC‑MS/MS-System injiziert. Nach chromatographischer Trennung werden Analyt und ISTD anhand der Retentionszeiten sowie der charakteristischen Massenübergänge identifiziert (siehe Tabelle 4). In jeder Analysenserie werden der Poolurin als Leerwert (S00- sowie S0-Kalibrierstandard) sowie Qualitätskontrollproben mitanalysiert.

Zur Überprüfung der Effizienz der enzymatischen Hydrolyse wird der Massenübergang des MU (siehe Tabelle 4) herangezogen. Bei erfolgreicher Hydrolyse sollte das Signal bei mindestens 2 × 10^6^ (Abundanz) liegen. Sollte ein deutlich kleineres bzw. kein Signal detektierbar sein, kann von einer unzureichenden Hydrolyse der betreffenden Probe ausgegangen werden und die Probe sollte erneut aufgearbeitet und analysiert werden.

## 8 Kalibrierung

Die nach Abschnitt 4.5 hergestellten Kalibrierstandards werden analog zu den Urinproben – ohne weitere Zugabe von ISTD – aufgearbeitet (siehe Abschnitt 5) und entsprechend den Abschnitten 6 und 7 analysiert. Die Kalibriergerade wird durch lineare Regression mit 1/y-Gewichtung der Peakflächenverhältnisse von EQI/EQI‑D_10_ gegen die Konzentrationen der jeweiligen Kalibrierstandards erstellt. Unter den beschriebenen analytischen Bedingungen verläuft die Kalibriergerade im Konzentrationsbereich von 0,03 bis 20 μg/l Urin linear. Proben, die höhere Analytkonzentrationen aufweisen, müssen nach Verdünnung mit hochreinem Wasser erneut analysiert werden. Bei der Berechnung der Analytkonzentration wird der entsprechende Verdünnungsfaktor berücksichtigt. Um zu überprüfen, ob Proben nach Verdünnung korrekt gemessen werden, wurden drei unterschiedliche Urine mit 30 μg EQI pro Liter dotiert und nach 1 ∶ 3-, 1 ∶ 6- sowie 1 ∶ 30-Verdünnung aufgearbeitet und analysiert. Je Urin und Verdünnung wurde eine Fünffachbestimmung durchgeführt, wobei die ermittelten Richtigkeiten innerhalb der Akzeptanzbereich von 85–115 % lagen.

**Abb. 3:**
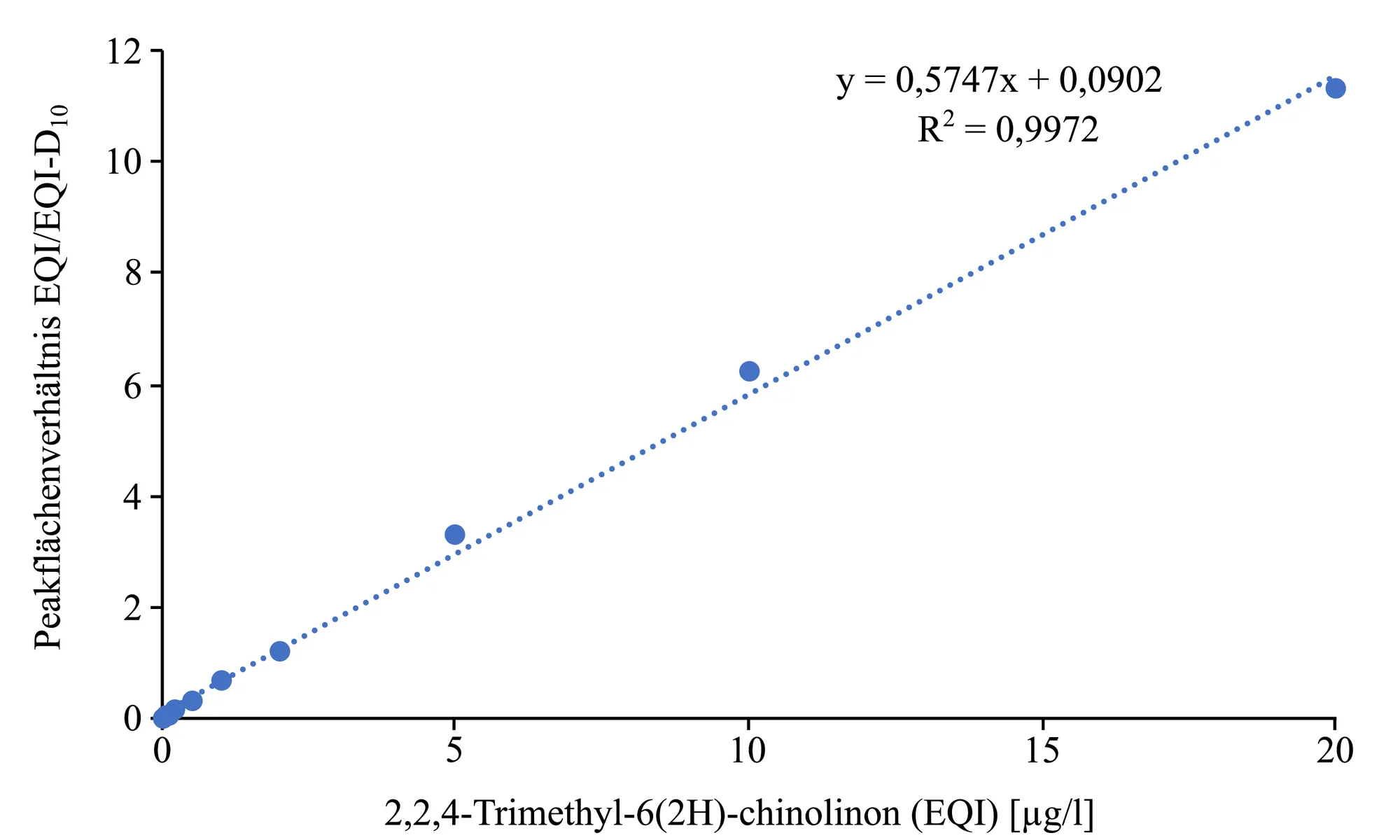
Kalibriergerade zur Bestimmung von EQI in Urin

Eine beispielhafte Kalibriergerade für die Bestimmung von EQI im Urin ist in Abbildung 3 dargestellt.

## 9 Berechnung der Analysenergebnisse

Die Berechnung der Analytkonzentration in der jeweiligen Urinprobe erfolgt mithilfe der zur Analysenserie gehörenden Kalibrierfunktion (siehe Abschnitt 8). Dabei wird das Peakflächenverhältnis von Analyt zu ISTD in die Kalibrierfunktion eingesetzt und die Analytkonzentration in μg/l Urin erhalten.

## 10 Standardisierung der Messergebnisse und Qualitätssicherung

Zur Sicherung der Qualität der Analysenergebnisse wird gemäß den Richtlinien der Bundesärztekammer und den Angaben in dem von der Kommission veröffentlichten allgemeinen Kapitel verfahren (Bader et al. 2010; Bundesärztekammer 2023).

Zur Präzisionskontrolle wird mit jeder Analysenserie mindestens eine Urinprobe mit bekannter Analytkonzentration als Qualitätskontrolle mitgeführt. Da ein derartiges Qualitätskontrollmaterial kommerziell nicht erhältlich ist, muss es selbst im Labor hergestellt werden. Dazu wird Humanurin mit einer definierten Menge an EQI dotiert, aliquotiert und bei −20 °C gelagert. Für die Validierung dieser Methode wurden vier verschiedene Kontrollmaterialien hergestellt: Q_1_ enthielt 0,03 μg EQI/l, Q_2_ 0,10 μg EQI/l, Q_3_ 1,00 μg EQI/l und Q_4_ 10,0 μg EQI/l.

## 11 Beurteilung des Verfahrens

Die Zuverlässigkeit des Verfahrens wurde durch eine umfassende Validierung sowie durch Nachstellung und Prüfung der Methode in einem zweiten, unabhängigen Labor bestätigt. Die Vorgehensweise bei der Methodenvalidierung (gemäß den Kriterien der DFG‑Arbeitsgruppe AibM (Bader et al. 2010) und der FDA-Guideline (FDA 2018)) sowie die Ergebnisse der Validierung sind nachfolgend beschrieben.

### 11.1 Präzision

Die Präzision in der Serie wurde unter Verwendung der Qualitätskontrollmaterialien (siehe Abschnitt 10) ermittelt, indem diese fünffach parallel aufgearbeitet und analysiert wurden. In Tabelle 5 sind die Daten zur Präzision in der Serie angegeben.

**Tab. 5:** Präzision in der Serie für die Bestimmung von EQI in Urin (n = 5)

Analyt	Dotierte Konzentration [μg/l]	Gemessene Konzentration [μg/l]	Standardabweichung (rel.) *s_w_* [%]	Streubereich *u* [%]
EQI	0,03	0,03	5,72	15,8
	0,10	0,11	0,51	1,42
	1,00	1,07	2,28	6,33
	10,0	9,95	1,71	4,75

Zur Ermittlung der Präzision von Tag zu Tag wurden die relativen Standardabweichungen aus den jeweiligen Mittelwerten von fünf parallelen Bestimmungen an drei Messtagen berechnet. Die so erhaltenen Präzisionsdaten sind in Tabelle 6 angegeben.

**Tab. 6:** Präzision von Tag zu Tag für die Bestimmung von EQI in Urin (n = 3)

Analyt	Dotierte Konzentration [μg/l]	Gemessene Konzentration [μg/l]	Standardabweichung (rel.) *s_w_* [%]	Streubereich *u* [%]
EQI	0,03	0,03	5,34	23,0
	0,10	0,11	3,53	15,2
	1,00	1,10	3,13	13,5
	10,0	10,29	3,44	14,8

### 11.2 Richtigkeit

Die relative Wiederfindung des Analyten wurde aus den Daten der Präzision von Tag zu Tag berechnet. Es ergaben sich die in Tabelle 7 aufgeführten relativen Wiederfindungen.

**Tab. 7:** Relative Wiederfindung für die Bestimmung von EQI in Urin (n = 3)

Analyt	Dotierte Konzentration [μg/l]	Mittlere Wiederfindung (rel.) *r* [%]	Bereich [%]
EQI	0,03	106	102–111
	0,10	107	106–108
	1,00	110	107–112
	10,0	103	99,5–107

### 11.3 Überprüfung der aufarbeitungsbedingten Verluste

Zur Überprüfung der aufarbeitungsbedingten Verluste wurden native Urinproben mit unterschiedlichen Hintergrundgehalten an EQI jeweils vor und nach der Probenaufarbeitung mit EQI in einer Konzentration von 0,10 μg/l, 1,00 μg/l und 10,0 μg/l dotiert. Die Proben wurden dreifach parallel aufgearbeitet und gemessen, woraus sich mittlere absolute Wiederfindungen von 13,3 %, 14,6 % und 17,1 % (Tabelle 8*)* ergaben. Diese eher geringen Wiederfindungen sind darauf zurückzuführen, dass zur Extraktion des Analyten aus ca. 3,5 ml wässriger Phase lediglich 0,4 ml Ethylacetat verwendet wurden.

**Tab. 8:** Mittlere absolute Wiederfindung für die Bestimmung von EQI in Urin (n = 3)

Analyt	Dotierte Konzentration [μg/l]	Mittlere absolute Wiederfindung [%]	Bereich [%]
EQI	0,10	13,3	11,3–14,7
	1,00	14,6	13,2–15,7
	10,0	17,1	16,8–17,3

### 11.4 Stabilität

Zur Überprüfung der Stabilität des Analyten wurden die Kurzzeitstabilität, die Frier-Tau-Stabilität sowie die Stabilität des Analyten nach Aufarbeitung untersucht. Für die Ermittlung der obengenannten Parameter wurden zwei Urine mit einer niedrigen sowie einer hohen EQI-Konzentration dotiert. Die Ergebnisse der Stabilitätsprüfung sind in Tabelle 9 aufgeführt. Die Kurzzeitstabilität des Analyten wurde nach einer Lagerdauer von ca. 20 h bei Raumtemperatur ermittelt, um Probenahme und Aufarbeitung widerzuspiegeln. Die Bestimmung der Frier-Tau-Stabilität erfolgte mit einem Urinaliquot, welches in Triplikaten direkt sowie jeweils nach sechs Frier-Tau-Zyklen aufgearbeitet und gemessen wurde. Die post-präparative Stabilitätsprüfung liefert einen Hinweis auf die Beständigkeit der messfertigen Extrakte unter realen Messbedingungen im Autosampler. Dafür wurden aufgearbeitete Proben, die für vier Tage im gekühlten Autosampler bei 10 °C standen, erneut analysiert. Für die EQI-Bestimmung wurden alle Akzeptanzkriterien der Stabilität (Richtigkeit zwischen 85 % und 115 % des Sollwerts) erfüllt.

**Tab. 9:** Ergebnisse der Stabilitätstests (n = 3)

Stabilitätsprüfung	Dotierte Konzentration [μg/l]	Gemessene Konzentration [μg/l]	Mittlere relative Wiederfindung [%]	Bereich [%]
Kurzzeitstabilität: 20 h bei RT	0,05	0,05	98,2	86,0–115
	12,17	10,81	88,8	88,4–89,1
Frier-Tau-Stabilität: nach 6 Zyklen	0,05	0,05	99,7	88,0–108
	12,17	12,46	102	97,5–106
Post-präparative Stabilität: 4 d bei 10 °C	0,05	0,04	86,3	85,1–87,2
	12,17	12,02	98,7	94,3–103

### 11.5 Nachweis- und Bestimmungsgrenze

Die Ermittlung der Bestimmungsgrenze (BG) erfolgte mittels Dotierung eines Poolurins, der an drei Tagen jeweils fünffach aufgearbeitet und gemessen wurde. Als Kriterien dienten die Richtigkeit sowie die Präzision der Bestimmungen. Für EQI lagen die Einzelwerte der Richtigkeit an der BG zwischen 80 % und 120 %, für die Präzision wurde ein Variations­koeffizient von 20 % nicht überschritten. Die BG wurde somit auf 0,03 μg EQI/l Urin festgelegt. Die Nachweisgrenze ergibt sich rechnerisch durch Division der BG durch drei zu 0,01 μg EQI/l Urin (Tabelle 10).

**Tab. 10:** Nachweis- und Bestimmungsgrenze für die Bestimmung von EQI in Urin

Analyt	Nachweisgrenze [μg/l]	Bestimmungsgrenze [μg/l]
EQI	0,01	0,03

### 11.6 Störeinflüsse

Während der Methodenentwicklung stellte sich heraus, dass das Ethoxyquin selbst in nicht aufgearbeiteten Urinproben innerhalb weniger Tage zu mehr als 50 % abgebaut wird. Darüber hinaus war Ethoxyquin in den Urinproben der Metabolismusstudie nur in geringen Konzentrationen nachweisbar. Dies hatte zur Folge, dass Ethoxyquin von den Methodenentwicklern als „nicht als Biomarker geeignet“ eingestuft wurde und die Methode nur für den Ethoxyquinmetaboliten EQI validiert wurde.

Stabilitätstests zeigten, dass EQI in Urinproben über sechs Frier-Tau-Zyklen stabil ist (siehe Abschnitt 11.4). Ein häufiges Auftauen und Einfrieren sollte vermieden werden. Bei der Probenaufarbeitung ist es erforderlich, den Proben vor der Hydrolyse Ascorbinsäure zuzusetzen (siehe Abschnitt 5.2), um die Stabilität des Analyten zu erhöhen.

Aufgrund der Flüchtigkeit des EQI konnte für die SALLE kein Lösungsmittel verwendet werden, das hätte abgeblasen werden müssen. Mit Ethylacetat wurde daher ein Lösungsmittel gewählt, das sich für die anschließende chromatographische Trennung eignete (Stoeckelhuber et al. 2020). Dabei führte das geringe Extraktionsmittelvolumen von nur 0,4 ml Ethylacetat zu erheblichen Analytverlusten während der Probenaufbereitung (siehe Abschnitt 11.3), die auch von den Prüfern der Methode beobachtet wurden. Durch die Verwendung eines strukturidentischen deuterierten ISTD konnten diese Verluste jedoch gut ausgeglichen werden ohne die Sensitivität der Methode maßgeblich zu beeinträchtigen.

Auch die bei der Methodenprüfung beobachtete Signalsuppression konnte durch den ISTD gut kompensiert werden. Da von den Methodenentwicklern keine Matrixeffekte beobachtet wurden, ist es denkbar, dass neben den modifizierten Chromatographiebedingungen während der Prüfung auch die von den Methodenprüfern verwendete *β*‑Glucuronidase/Arylsulfatase aus *Helix pomatia* eine Rolle gespielt haben könnte (siehe auch Stoeckelhuber et al. 2020).

Von den Entwicklern der Methode wurde nach Injektion von Proben mit hohen Analytkonzentrationen ein Carry-over-Effekt in Höhe der BG gefunden. Daher sollten nach hohen Kalibrierstandards und Qualitätskontrollproben Leerwertproben injiziert werden. Wenn in Proben, die in einer Sequenz Proben mit hohen Analytgehalten folgen, EQI-Gehalte um die BG gemessen werden, so müssen diese reinjiziert werden.

Für den Fall, dass die Überprüfung der enzymatischen Spaltung eine unzureichende Hydrolyseeffizienz ergibt, empfiehlt es sich, die betreffende Probe erneut aufzuarbeiten und zu analysieren (siehe Abschnitt 7).

## 12 Diskussion der Methode

Ziel der Methodenentwicklung war die Erarbeitung einer schnellen und selektiven Analysenmethode, um eine mögliche Exposition der Allgemeinbevölkerung gegen Ethoxyquin zu erfassen (Stoeckelhuber et al. 2020). In dieser Methodenvorschrift wird nicht nur die entwickelte UPLC‑MS/MS-Methode für die Bestimmung von EQI in Urin vorgestellt, sondern auch detailliert auf die dabei erhobenen Validierungsdaten eingegangen und die Ergebnisse aus der umfassenden Methodenprüfung in einem zweiten, unabhängigen Labor dargestellt. Die Methode hat sich als sensitiv und präzise erwiesen und besitzt eine gute Richtigkeit. Die BG ist ausreichend, um den Ethoxyquinmetaboliten EQI in den meisten Urinproben der Allgemeinbevölkerung zu quantifizieren und damit auch die nicht-berufliche Exposition gegen Ethoxyquin abzuschätzen.

**Verwendete Messgeräte** UPLC‑MS/MS-System bestehend aus den Modulen LC‑20AB (Hochdruckpumpe), LC‑30AD (Ultradruckpumpe), CBM‑20A (System-Controller), DGU‑20A5R (Degasser), CTO‑20AC (Säulenofen) und SIL‑30ACMP (Autosampler) (Shimadzu Deutschland GmbH, Duisburg) sowie einem QTRAP^®^ 6500+ Tandem-Quadrupol-Massenspektrometer (AB SCIEX Germany GmbH, Darmstadt)
